# In vitro evidence of decompression bubble dynamics and gas exchange on the luminal aspect of blood vessels: Implications for size distribution of venous bubbles

**DOI:** 10.14814/phy2.14317

**Published:** 2019-12-25

**Authors:** Ran Arieli

**Affiliations:** ^1^ The Israel Naval Medical Institute Israel Defense Forces Medical Corps Haifa Israel; ^2^ Institute for Medical Research Galilee Medical Center Nahariya Israel

**Keywords:** active hydrophobic spot, blood vessel, gas micronuclei, nanobubbles

## Abstract

We found that lung surfactant leaks into the bloodstream, settling on the luminal aspect of blood vessels to create active hydrophobic spots (AHS). Nanobubbles formed by dissolved gas at these AHS are most probably the precursors of gas micronuclei and decompression bubbles. Sheep blood vessels stretched on microscope slides, and exposed under saline to hyperbaric pressure, were photographed following decompression**.** Photographs of an AHS from a pulmonary vein, containing large numbers of bubbles, were selected in 1‐min sequences over a period of 7 min, starting 18 min after decompression from 1,013 kPa. This showed bubble detachment, coalescence and expansion, as well as competition for dissolved gas between bubbles. There was greater expansion of peripheral than of central bubbles. We suggest that the dynamics of decompression bubbles on the surface of the blood vessel may be the closest approximation to true decompression physiology, and as such can be used to assess and calibrate models of decompression bubbles. We further discuss the implications for bubble size in the venous circulation.

## Graphical abstract

Bubble formation on luminal aspect of blood vessels are the best experimental model for decompression bubbles, their interactions and dynamics.

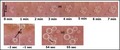

## 1 INTRODUCTION

We have proposed that decompression illness is related mainly to bubbles that expand from nanobubbles on the luminal aspect of venous blood vessels (Arieli, 2017). On detachment, the bubbles tear off pieces of the endothelium, resulting in endothelial injury. Venous bubbles may shunt to the arterial circulation, whereas others, after losing their gas in the lungs, are left as microparticles that induce platelet and neutrophil activation. Bubbles which expand and develop from nanobubbles on the surface of distal arteries may be the cause of Taravana, micro‐injuries in the white matter, vestibular, and spinal decompression illness (DCI) (Arieli, 2019a, 2019b; Arieli & Marmur, 2017). Extravascular hydrophobic surfaces within the body provide the background for the remaining forms of decompression illness (joint pain, cutis marmorata, osteonecrosis, and spinal DCI (Arieli, 2018a, 2018b).

Vast amounts of literature have been devoted to the attempt to establish the source and stability of gas micronuclei as the precursors of decompression bubbles. The production and stability of nanobubbles on a hydrophobic surface presented a solution to the long sought‐after gas micronuclei from which decompression bubbles develop. We have demonstrated that surfactant leaks from the lung into the bloodstream, settling on the luminal aspect of blood vessels to create active hydrophobic spots (AHS). Nanobubbles formed by dissolved gas at these AHS are most probably the precursors of gas micronuclei and decompression bubbles. In previously reported studies (Arieli, Arieli, & Marmur, 2015; Arieli & Marmur, 2016), sheep blood vessels stretched on microscope slides, and exposed under saline to hyperbaric pressure, were photographed at 1‐s intervals following decompression. The methods employed, as well as the measured bubble size on detachment and their rate of expansion, were described in the above mentioned reports. The frequency of photography enabled close observation of the interaction between bubbles and supersaturated, well‐mixed saline following decompression.

There is also an ample body of literature dealing with calculation of the physics and dynamics of decompression bubbles, however without any experimental evidence of the formation and behavior of bubbles within the body. Our observation of decompression bubbles on the luminal aspect of blood vessels, with our suggestion that the nucleation of nanobubbles on hydrophobic surfaces within the body—veins, distal arteries, and extravascular sites, may be the direct cause of DCI, give me reason to believe that this is at present the closest approximation to the true physiology of decompression illness. In this study, we discuss solid grounds for a further analytical approach, and the implications for the size distribution of venous bubbles.

## 2 METHODS AND RESULTS

**Figure 1:**
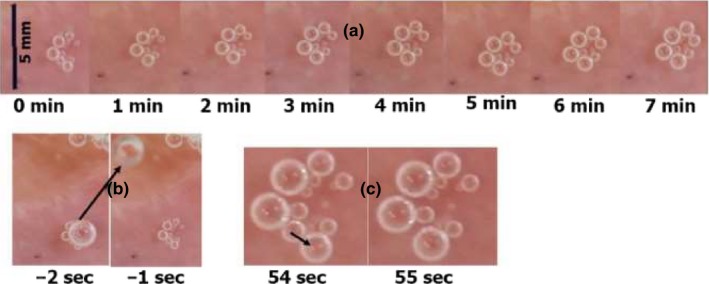
(a) Photographs of bubbles at an active hydrophobic spot (AHS) on a pulmonary vein, over a period of 7 min, starting 18 min (time 0) from decompression. (b) Detachment of a large bubble (arrow) 1 s before time 0. (c) Coalescence of two bubbles (indicated by the arrow). The sudden increase in volume of the single bubble formed can be seen in the frame on the right

A detailed description of the data collection methods may be found in our previous study (Arieli et al., 2015; Arieli & Marmur, 2016). Photographs of an AHS from a pulmonary vein, containing large numbers of bubbles, were selected in 1‐min sequences over a period of 7 min (Figure 1a). The first photograph was taken 18 min after decompression from 1,013 kPa, following detachment of a large bubble from this AHS (Figure 1b). The initial gas tension in the saline was calculated to be 620 kPa, and gas was released from the saline to the atmosphere by means of a peristaltic pump, which continuously recirculated the supersaturated saline. In general, bubbles expanded from the dissolved gas with time. The two lowermost bubbles at time 0 coalesced into one bubble (at time 1 min), as depicted in Figure 1c. When a bubble disappeared, the neighboring bubble was seen immediately to expand. Bubbles on the periphery expanded faster than bubbles at the center. The two small peripheral bubbles at the open right side of the cluster (time 0) expanded faster than similarly sized central bubbles (at time 7 min). In no case did we see a small bubble decrease in size, as may have been expected if small bubbles transferred their gas to larger bubbles due to differences in gas tension. The contact area of the bubble with the blood vessel wall is small, as may be seen from the area left by the detached bubble at time –1 s (Figure 1b), and from the small bubbles visible underneath the large one at time –2 s (Figure 1b). A side view of the bubbles formed on hydrophobic silicon wafers showed that they were spherical (Arieli & Marmur, 2013), which also concurs with their having a small contact area.

## 3 DISCUSSION

### 3.1 Bubble dynamics

The spherical form of bubbles at the AHS implies that most of the gas exchange in a bubble takes place with the surrounding medium (blood), and not with the tissue across the vessel wall. Small bubbles with a high internal pressure (the Laplace equation) and high gas tension would have been expected to transfer gas to larger bubbles having a lower gas tension (Ostwald, 1897). Thus, small bubbles would shrink and disappear, and in equilibrium all the bubbles would be of equal size. This effect, known as "broadening", has been applied to several analyses of decompression bubbles in the past, and also more recently (Hills, 1977, 1978; Wienke, O'Leary, & Del Cima, 2018). However, this mechanism was not observed in our experiments.

Competition for the dissolved gas among bubbles growing in close proximity to one another was examined in theoretical studies by Karapantsios, Kostoglou, Divinis, & Bontozoglou (2008) and by Van Liew & Burkard (1985). This effect can clearly be seen in this study, with bubbles on the periphery expanding much faster than those at the center. Thus, even with a well‐mixed medium, peripheral bubbles "steal" the gas in the medium from central bubbles. Coalescence of bubbles was discussed by Hills (1977) for both stationary bubbles and bubbles in the blood, and is seen clearly in the example presented here. Coalescence has been blamed in the past for the delayed joint pain of decompression illness (Hills, 1977). More recently, I presented an alternative explanation for joint pain (Arieli, 2018a). These types of observation can be used to assess and calibrate mechanical models of decompression bubbles.

### 3.2 Size distribution

**Figure 2:**
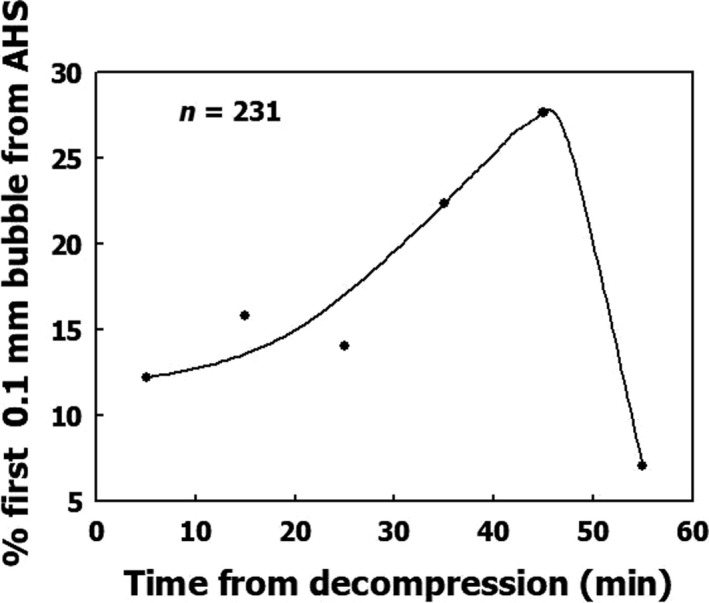
Time distribution of the first bubble to reach a diameter of 0.1 mm at each AHS. The line is hand drawn

The time to the first appearance of a bubble at an AHS is varied. In Figure 2, I compiled data from our previous study (Arieli & Marmur, 2016) to present the time distribution for the first observation of a bubble with a diameter of 0.1 mm at each AHS. Few AHS are initiated within the first quarter of an hour after decompression, many are initiated between half‐an‐hour to 40 min, and a few toward the end of the hour. Therefore, surface nanobubbles, which adhere to the AHS, will differ in size within any given time range.

**Figure 3:**
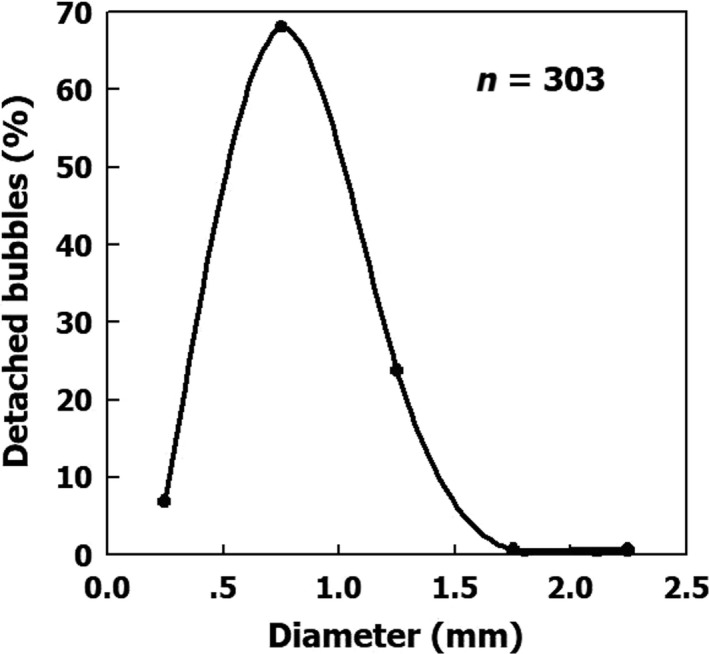
Percentage of detached bubbles as a function of their diameter

Bubbles detached from the blood vessels in the experimental setup, most probably when buoyancy equaled the intermembrane adhesion force (Arieli, 2017). This yielded a rather homogeneous size distribution, as shown in Figure 3 by the data compiled from the previously reported study (Arieli et al., 2015). Most of the bubbles detached with a diameter of 0.5‒1.5 mm, and very few with a diameter of <0.5 or 1.5‒2.5 mm. In this case, however, detachment was from large, flattened blood vessels. The in vivo conditions within narrow vessels would not allow bubbles to expand to that size, so that with their smaller diameter they could then easily be carried in the bloodstream. Other phenomena such as turbulence, physical movement, and any additional disturbances will also drive small bubbles from the AHS into the bloodstream. In our experimental setup, the pulsatile flow was greater than that in most blood vessels, but less than that in the large vessels. Therefore, faster flow in large blood vessels may drive bubbles that are smaller than our measured bubbles. The increased Doppler detection of venous bubbles following exercise is due to early release of bubbles from the AHS into the bloodstream. Therefore, bubbles postexercise may be smaller in comparison with those observed in resting conditions. This may serve as an explanation for the increased risk of DCI when exercise follows decompression. It is safer to allow the surface bubbles to lose their gas at the AHS where they are formed.

In conclusion, some of the currently used algorithms for the construction of decompression tables, such as the varying permeability model, based as it is on the theoretical considerations of Yount and Hoffman (1986), are founded on the size distribution of microbubbles in gelatin. Others, such as the reduced gradient bubble model (Wienke, 1990), have developed theories of an exponential distribution of gas micronuclei. To date, the in vitro experimental setup presented here appears to be the best system for studying the dynamics of decompression bubbles, and may be used to develop and calibrate theoretical models of decompression bubbles.

## CONFLICT OF INTEREST

No conflicts of interest, financial or otherwise, are declared by the author.

## AUTHOR CONTRIBUTIONS

R.A. drafted manuscript, edited and revised, and approved final version of manuscript.
